# Liquid-Based Screening Tests Results: HPV, Liquid-Based Cytology, and P16/Ki67 Dual-Staining in Private-Based Opportunistic Cervical Cancer Screening

**DOI:** 10.3390/diagnostics11081420

**Published:** 2021-08-05

**Authors:** Martyna Trzeszcz, Maciej Mazurec, Robert Jach, Karolina Mazurec, Zofia Jach, Izabela Kotkowska-Szeps, Magdalena Kania, Mariola Wantuchowicz, Anna Prokopyk, Piotr Barcikowski, Marcin Przybylski, Joanna Wach, Agnieszka Halon

**Affiliations:** 1 Corfamed Woman’s Health Center, Kluczborska 37, 50-322 Wroclaw, Poland; carlos.mazurec@icloud.com (K.M.); iza.corfamed@gmail.com (I.K.-S.); magda_kania@poczta.onet.pl (M.K.); mariolawantuchowicz@gmail.com (M.W.); ania.prokopyk@gmail.com (A.P.); barcikowski07@gmail.com (P.B.); asia03529@gmail.com (J.W.); 2 Division of Pathology and Clinical Cytology, University Hospital in Wroclaw, Borowska 213, 50-556 Wroclaw, Poland; 3 Division of Gynecologic Endocrinology, Jagiellonian University Medical College, Kopernika 23, 31-501 Krakow, Poland; jach@cm-uj.krakow.pl; 4 Superior Medical Center, Czyzynska 21/50, 31-571 Krakow, Poland; zofiajach@gmail.com; 5 Department of Obstetrics and Gynecology, District Public Hospital, Juraszow 7-19, 60-479 Poznan, Poland; nicramp@poczta.onet.pl; 6 Department of Practical Obstetrics, Wroclaw Medical University, Bartla 5, 51-618 Wroclaw, Poland; 7 Department of Clinical and Experimental Pathology, Division of Clinical Pathology, Wroclaw Medical University, Borowska 213, 50-556 Wroclaw, Poland; agnieszka.halon@umed.wroc.pl

**Keywords:** cervical cancer screening, high-risk HPV testing, cervical cytology, p16/Ki67 dual-staining, prevention and control

## Abstract

The baseline data from the private-based opportunistic cervical cancer screening with HRHPV14, liquid-based cytology (LBC) and p16/Ki67 testing, and its quality assessment/quality control (QA/QC) tools are lacking. The age-stratified analysis of 30,066 screening tests results in a Polish population, including the investigation of HRHPV14 status, LBC, and p16/Ki67 dual-staining reporting rates, along with immediate histopathologic correlations, was conducted. For cytopathologic QA/QC, the College of American Pathologists (CAP) benchmarks and enhanced safety protocol were used. The NILM/ASC-US/LSIL/ASC-H/HSIL/AGC reporting rates were 93.9/3.4/2.0/0.22/0.24/0.11, respectively, with correlating HRHPV14-positive rates of 8.4/48.9/77.2/84.6/90.7/26.7. The reporting rates for HSIL (CIN2+) in HRHPV-positive women with NILM/ASC-US/LSIL/ASC-H/HSIL/AGC referred for a colposcopy with biopsy were 19.1/25.8/22.5/12.4/19.1/1.1% of the total HSIL (CIN2+). In total, of the 1130 p16/Ki67 tests, 30% were positive. In NILM HRHPV14-positive women with available histology result, HSIL(CIN2+) was detected in 28.3% of cases. In the first such large-scale Polish study presenting HRHPV14, informed LBC and HSIL (CIN2+) results, the reporting rates were highly consistent with data from American and other CAP-certified laboratories, confirming the possibility of using the 2019 ASCCP risk-based guidelines as one of the screening strategies outside of the US, in conditions of proper QA/QC. The private-based screening model can be effective in cervical cancer prevention, particularly in countries with low population coverage of public funds-based systems.

## 1. Introduction

HPV testing is globally recommended as a primary screening strategy in cervical cancer prevention in all resource settings [1]. According to the European guidelines, it should be implemented under the population-based organized system, which is currently the most effective model of screening [2].

In Poland, a Central Eastern Europe country, two main models of cervical cancer screening have been coexisting for years: a public funds-based and a private-based model. In the years 2006–2016, the only organized population-based national program with primary cytology screening in 3-year screening intervals was implemented [3,4,5]. However, coverage of the target group in the program did not exceed 27% [6,7], which does not meet the recommended 70% population coverage needed to reach screening effectiveness [2]. In parallel to the public funds-based screening in Poland, there is a private-based screening financed directly by the patients. Its exact screening coverage remains unknown, but published questionnaire data suggest a significant percentage of involvement. In 2012, the participation of Polish women in screening outside of the program was 1.7 times higher than in the program [4,8], and in the last 3 years, about 70% of women have had a Pap test [9], which clearly suggests the dominating role of the private-based screening. There are various models in Polish private-based screening, from cytology to cotesting; the latter was approved in 2013 [10].

Due to the high sensitivity and high specificity of p16/Ki67 dual-staining for the detection of HSIL (CIN2+) [11,12,13], it was introduced in Poland for triaging patients with ASC-US or LSIL in 2016 [14]. The effectiveness of the extended screening strategy in the HPV-based model with p16/Ki67 testing has been confirmed by FDA registration [15]. The limitations of the Polish population-based screening, the preferences of Polish women, and the growing awareness of private sector gynecologists in the new paradigms of secondary cervical cancer prevention result in the gradual implementation of the globally recommended HPV-based screening strategies with proven higher effectiveness [2,16] in Polish private gynecological care [17,18]. Interim screening guidelines in the SARS-CoV-2 pandemic of 2021 [18] include the 2019 risk-based management guidelines of the American Society of Colposcopy and Cervical Pathology (ASCCP) [16] as one of the screening options. The 2019 ASCCP guidelines indicate that women with the same screening tests results, together with a similar screening history, have a similar CIN3+ risk, regardless of geographic location, race, ethnicity, or socioeconomic status.

We conducted a retrospective analysis of liquid-based screening (LBS) tests results, including liquid-based cytology (LBC), 14 types of high-risk human papilloma virus (HRHPV14), and p16/Ki67 HSIL-risk biomarkers, along with histologic correlations in private-based opportunistic cervical cancer screening. This is the first Polish investigation on such a large scale. The second objective of the study was a comparative analysis of the obtained cytologic and virologic reporting rates with the American data for gynecological cytopathology, in order to verify the effectiveness of the quality assessment and quality control (QA/QC) tools used, confirming that it is possible to apply the 2019 ASCCP guidelines in private-based opportunistic screening outside the US. Our study also addresses how to open the way for an integration of organized population-based and opportunistic private-based models into a co-existing hybrid model, giving the opportunity to achieve the required population screening coverage in Poland and in other countries with similar economic-cultural conditions.

## 2. Materials and Methods

### 2.1. Study Population

This study is a post-hoc analysis on LBS, including LBC, HRHPV14, and p16/Ki67 tests results, obtained in private-based opportunistic screening in one of the largest private outpatient gynecological clinics in an urban area of the Lower Silesia in Poland (Corfamed Woman’s Health Center) from August 2015 to July 2020. This age-stratified analysis comprises a total of 30.066 screening tests. Three cervical cancer screening models have been applied in the Center during the analyzed period: primary cytology with reflex HPV testing, primary cotesting including simultaneous performance of HPV testing and cytology, and primary cotesting plus (cotesting with simultaneous p16/Ki67 testing). In the primary cytology screening, the reflex HRHPV14 test was recommended in all cases with minor cytological abnormalities (ASC-US or LSIL). The p16/Ki67 test was performed in cytology- and cotesting-based models, in cases with ASC-US or LSIL in cytology, and for all positive HRHPV14 test results. The retrospective analysis of cytologic-virologic-immunocytochemical tests results, along with the histology results obtained from colposcopies performed at the Center, were the endpoints of this study. All analyzed data were from the Centre’s registry.

### 2.2. HRHPV Testing

For HRHPV detection, the Abbott RealTime High Risk HPV molecular in vitro PCR test was used. A laboratory performing the test declared that it was in accordance with the manufacturer’s protocol. The test phenotypes the DNA of 14 highly oncogenic HPV types (16, 18, 31, 33, 35, 39, 45, 51, 52, 56, 58, 59, 66, and 68) with HPV 16 and 18 genotyping. All screening tests (HRHPV14, LBC and p16/Ki67) were performed in the same laboratory.

### 2.3. Cytology

All cervical cytology was collected by the Center’s gynecologists using the Cervex-Brush device (Rovers Medical Devices, Oss, The Netherlands) and transferred on the detachable head into the SurePath vial containing a preservative fluid. Cervical sampling was carried out with rotation of the broom in a clockwise direction five times, after inserting the central bristles into the endocervical canal. The SurePath liquid-based preparation was performed in accordance with the automated PrepStain procedure (Becton Dickinson, Franklin Lakes, NJ, USA) by an external laboratory, which ensured that its performance strictly adhered to a manufacturer’s protocol. All cytology samples were reported by a gynecological cytopathologist in the Bethesda 2014 system. A pathologist was employed by the Center and was informed about HRHPV14 status. All residual cervical samples were stored for up to 6 months by the laboratory under the conditions specified by the manufacturer. It provided no need to call the patient for another visit when an ancillary testing was ordered (including the p16/Ki67).

### 2.4. P16/Ki67 Dual-Stain Testing

For immunocytochemical double staining with p16 and Ki67 proteins, a CINtec PLUS detection kit (Roche, MTM AG laboratories, Munich, Germany) was used and carried out in an automated BenchMark XT system (Ventana Medical Systems, Inc., Oro Valley, AZ, USA) according to the manufacturer’s protocol, as provided by the coordinator from the laboratory. The p16/Ki67 test was performed from the residual LBC material in the original SurePath vials (Becton Dickinson, Franklin Lakes, NY, USA), stored in a laboratory after cytology and/or HPV testing. The p16/Ki67 tests were evaluated by a gynecological pathologist (the same pathologist as for the LBC evaluation), specially trained and certified in p16/Ki67 test review, and reported as positive/negative/unsatisfactory in accordance with the available definitions [19].

### 2.5. Basic and Extended QA/QC Protocols

Gynecologic cytopathology QA/QC tools for morphologic evaluation in LBC preparation have not yet been standardized in Polish conditions, hence the need for the Center to rely on the available CAP QA/QC protocols. We modified the CAP protocol due to the p16/Ki67 testing used in this study, and the Center’s own original protocol for LBS tests results, including LBC, p16/Ki67 and HRHPV14, was created. Two protocols have been standardized in the study: (1) basic, the continuous quality improvement (CQI) protocol; (2) extended, the enhanced safety (ES) protocol. The basic protocols were used for the entire study group of 20,605 LBC, and the extended protocol for was used for the first 8330 LBC diagnoses.

#### 2.5.1. QA/QC Basic Protocol

In the internal QA/QC for LBC, a re-evaluation of 100% of ASC-US+ results (ASC-US or worse) and 100% of HRHPV14-positive NILM cases was performed, as well as ASC-H, HSIL/HRHPV14-negative, and NILM HRHPV14 type 16 and/or 18-positive cases. The re-evaluation of the LBC diagnoses was performed by one cytopathologist, the initial LBC results were blinded. Additionally, the CAP QA/QC gynecological cytopathology tools were implemented. For this purpose, obtained percentage rates in individual categories of cytological diagnoses were compared to the ranges reported by the CAP-certified laboratories for SurePath preparation [20,21]. The comparative reports were initially carried out in the period from August 2015 to March 2017, at 3 months intervals. After confirming the good quality of the cytopathological assessment, the intervals were extended to 12 months. The basic protocol also included the percentage rates estimation of HRHPV14-positive results within all cytologic diagnoses. For the cytologic-histologic correlations (CHC), the CQI protocol was based on bi-directional scenarios published by the 2012 CAP gynecologic cytopathology consensus conference [22]. The performed review included the percentage of concordant and discrepant diagnoses, as well as the positive predictive value (PPV) of a positive cytology result, which is the preferred standard CHC metric for gynecologic cytopathology [22].

Table 1 and Table 2 present the overview of correlating pairs used in the cytologic-immunocytochemical-histologic correlations (CICHC) assessment in our study. Table 1 shows positive CHC, defined as correlations of abnormal cytology or positive p16/Ki67 with positive histopathology (LSIL(CIN1)/HSIL(CIN2)/HSIL(CIN3)/AIS or cervical cancer). Table 2 shows a negative CICHC, defined as any abnormal cytology or positive/negative p16/Ki67 result that corresponds to a negative histopathological result and vice versa. Each diagnosis in the left column refers to each diagnosis in the right column in the Table 1 and Table 2.

#### 2.5.2. QA/QC Extended Protocol

To maximize the diagnostic-therapeutic safety, in the initial period of the study, the enhanced safety protocol was used, including the CQI protocol extended with an additional quality control procedure for the LBC interpretation. After receiving the batch of LBC slides, a cytopathologist performed rapid prescreening (RPS) with the turret method, that is a quick (not exceeding one minute) morphological review [23]. During RPS, slides were classified into one of 3 groups: suspicious, negative, and unsatisfactory. At this stage, a cytopathologist did not use the clinical data and the RPS results were not used until the routine screening was completed. The routine screening was performed as usual [24]. The third stage of cytological evaluation was RPS, and determining the routine screening results’ correlation with re-evaluation of all abnormal, suspicious, and unsatisfactory LBC. The final cytological diagnosis was established after the third stage was completed. RPS and re-evaluation of 100% abnormal LBC were performed after routine screening, using internal QA/QC tools in our ES protocol, which are recommended as the most effective for gynecologic cytopathology among various retrospective and prospective screening methods [23].

In both protocols (basic and extended), all LBC slides interpreted as unsatisfactory were prepared again at the request of the center, and the diagnostic procedure was repeated in its entirety.

### 2.6. Colposcopic Biopsy and Histology

The cases which were p16/Ki67-positive, ASC-US or LSIL HRHP14-positive, or with cytologic ASC-H or HSIL, regardless of HRHPV14 status, in accordance with the Polish recommendations with extension to the 2012 and 2015 ASCCP guidelines, were referred for a colposcopy with biopsy, performed by Center’s gynecologists [3,10,25,26]. The minimal colposcopy protocol requirements included endocervical sampling, direct biopsies for all cases if abnormal colposcopic findings (or suspicious for invasion) were identified (based on 2011 IFCPC nomenclature), and random biopsies when no abnormal colposcopic findings were found and new-SCJ was visible. The histologic diagnoses of cervical biopsies and endocervical curettages with brushings were reviewed according to LAST 2012 and WHO/IARC 2014 terminology by a gynecological pathologist employed by the Center [27,28]. Colposcopies performed outside the Center, and/or histologic reports interpreted by someone other than Center pathologists, were not included in the study due to different colposcopy protocols and/or different histologic nomenclature and/or morphologic criteria used. The reevaluation of cytology, histology, and colposcopic findings was always performed when cyto-immunocytochemical-colposcopic-histologic discrepancies occurred, including ASC-H and HSIL in cytology with negative or LSIL (CIN1) biopsy results, and were made in direct or phone/online multidisciplinary team meetings.

## 3. Results

### 3.1. LBS Reporting Rates

The age range of women included in the study was 15 to 92 years, with an average of 40.9 years, and the other group characteristics were: middle and higher economic status, mostly (>95%) from urbanized areas, and at least a secondary level of education. The total number of screening tests performed in the analyzed 5-year period was 30.066, including 20.605 cases of LBC reports, with 8331 HRHPV14 and 1130 p16/Ki67 tests available based on standardized indications [10,14,25,26] or at the request of patients (as cotesting plus test). The number and type of test performed (LBC, HRHPV14, and p16/Ki67) in subsequent years are listed in Table 3. The number of tests performed in individual age groups is presented in Table 4. Most LBC, HRHPV14, and p16/Ki67 testing was performed in the 30–39 age group.

In the analyzed population, the overall NILM, ASC-US, LSIL, ASC-H, HSIL, and AGC reporting rates were 93.9, 3.4, 2.0, 0.22, 0.24, and 0.11%, respectively. The percentage of HRHPV14 tests results was 37.7, 82.8, 87.6, 84.8, 87.8, and 65.2%, respectively, for the indicated above cytological diagnoses. The percentage of HRHPV14-positive tests results was 8.4, 48.9, 77.2, 84.6, 90.7, and 26.7%, respectively, for the corresponding cytological diagnoses. The percentage of p16/Ki67 tests results for the indicated cytological diagnoses was 2.4, 46.8, 65.0, 67.4, 69.4, and 34.8%, respectively. The percentage of p16/Ki67-positive tests results for the indicated cytological diagnoses was 17.4, 30.0, 35.2, 87.1, 91.2, and 62.5%, respectively (Table 5).

Age-stratified NILM, ASC-US, LSIL, ASC-H, HSIL, and AGC reporting rates were analyzed in eight age groups and all of the results are presented in Table 6. The NILM, ASC-US, LSIL, ASC-H, HSIL, and AGC reporting rates were highest in the group (age group No (%)): 7 (96.6), 3 (4.1), 1 (6.6), 5 (0.35), 7 (0.36), and 5 (0.26), respectively.

### 3.2. Histologic Correlation for LBS Results

The final analysis included cases with a performed colposcopy with biopsy within 3 months of obtaining the initial screening tests results. The cyto-histologic correlation results are summarized in Table 7. Of 20.605 cases, 415 (2.0% of all study groups) with LBC reports had a colposcopy with a biopsy procedure in our Center, from a total of 846 women referred for a colposcopy. A total of 93 cases of HSIL (CIN2+) were diagnosed, which was 22.4% of the total with biopsy results. No cases of SCC or AIS were identified. The highest number of HSIL (CIN2+) diagnoses (23 cases) were found among ASC-US cytology diagnoses, which was 5.5% of the total biopsy results. In other cytological diagnoses, it was reported as follows (No of detected HSIL (CIN2+) cases; % of total biopsy results): for NILM (17; 4.1%), LSIL (21; 5.1%), ASC-H (11; 2.7%), HSIL (20; 4.8%), AGC (1; 0.24%), SCC/AIS (0.0%). The following histological diagnoses were not included in the final analysis: B-DLBCL NOS (1 case), CGIN (1 case), SIL (3 cases), and HG CGIN (1 case).

The cyto-virologic-histologic correlations (CVHC) results are summarized in Table 8. Of 20.605 cases, 402 (2.0% of all study group) with LBC and HRHPV14 results had a colposcopy with a biopsy procedure in our Center. Negative and LSIL (CIN1) histology results were found in 76.4% of all HRHPV14-positive cases and in 92.0% of HRHPV14-negative cases. The positive histology results (HSIL (CIN2+)) in 97.8% (91 cases) were HRHPV14-positive, and in 2.2% (2 cases), HRHPV14-negative (both HSIL in cytology). In the study group, 17 cases of HSIL (CIN2+) NILM HRHPV14-positive were found, which was 18.7% of all HSIL (CIN2+) diagnoses.

The cyto-virologic-immunocytochemical-histologic correlations (CVICHC) results are summarized in Table 9. Of 20.605 cases, 375 (1.8% of all study group) with LBC, HRHPV14 and p16/Ki67 test results had a colposcopy with a biopsy procedure in our Center. Negative and LSIL (CIN1) histology results were found in 75.9% of all HRHPV14-positive cases and in 91.3% of HRHPV14-negative cases. The positive histology results (HSIL (CIN2+)) in 94.1% (80 cases) tested p16/Ki67-positive, and in 5.9% (5 cases) were p16/Ki67-negative in the HRHPV14-positive group. In the study group, 2 HSIL (CIN2+) cases which were p16/Ki67-positive and HRHPV14-negative were found, which was 100% of all HSIL (CIN2+) HRHPV14-negative results.

### 3.3. Basic and Extended QA/QC Protocols Results

The CQI protocol was used throughout the study and remained the basic QC in the Center protocol at the end of the study. Its full application with PPV evaluation for cytology results was possible from the 4th QC round. The ES protocol was used in the study until July 2017 for QC Round 6. The confirmation of the statistical compliance of both protocols in the subsequent QC rounds (1–6) allowed for a departure from the labor-intensive and time-consuming additional procedure without losing the quality of the cytopathological assessment. In total, 9 QC rounds were performed during our study. The first 5 rounds were performed, on average, every 3 months. Rounds 6 to 9 were performed in periods not exceeding 12 months. The next rounds, together with the increasing number of LBC cases assessed, are listed in Table 10.

**Figure 1:**
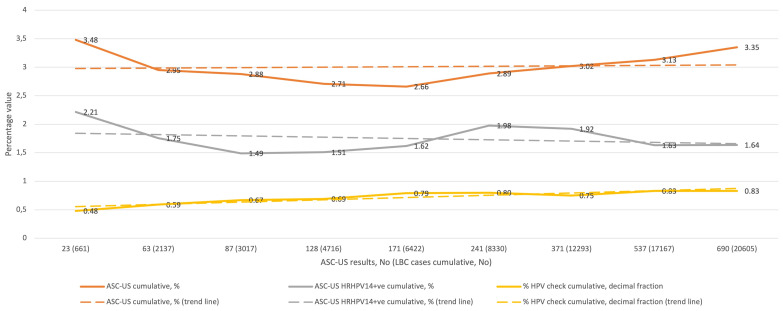
QC results for ASC-US in the study period (1–9 rounds). Abbreviations: QC, quality control; ASC-US, atypical squamous cells of undetermined significance; LBC, liquid-based cytology; HRHPV14, 14 high-risk types of human papillomavirus test; +ve, positive; HPV, HRHPV14.

Figure 1 shows the cumulative percentage of ASC-US diagnoses in relation to all LBC diagnoses (upper curve) in the subsequent QA/QC rounds, the cumulative percentage of HRHPV14-positive ASC-US diagnoses in relation to all LBC diagnoses (middle curve), and the cumulative percentage of HRHPV14-positive ASC-US diagnoses (presented as a decimal) over all ASC-US diagnoses (lower curve), and the corresponding trend curves. The lowest cumulative percentage of ASC-US diagnoses was 2.66% and the highest was 3.48%, and all values were between the 10th and 50th percentile reported by CAP-accredited laboratories for SurePath LBC preparation. The cumulative percentage of HRHPV-positive ASC-US for the entire study was 1.64% of all the LBC diagnoses, and for ASC-US diagnoses it was 48.86%.

**Figure 2:**
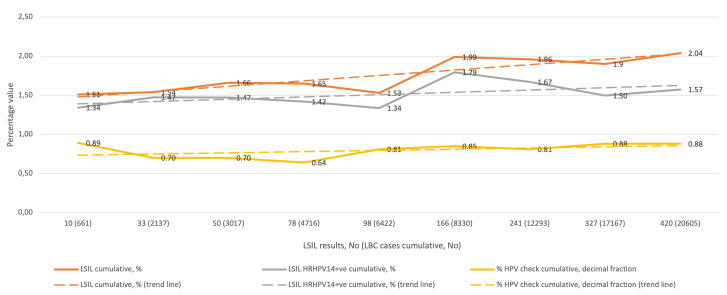
QC results for LSIL in the study period (1–9 rounds). Abbreviations: QC, quality control; LSIL, low-grade squamous intraepithelial lesion; LBC, liquid-based cytology; HRHPV14, 14 high-risk types human papillomavirus test; +ve; positive; HPV, HRHPV14.

Figure 2 shows the cumulative percentage of LSIL diagnoses in relation to all LBC diagnoses in the subsequent QA/QC rounds (upper curve), the cumulative percentage of HRHPV14-positive LSIL diagnoses in relation to all LBC diagnoses (middle curve), and the cumulative percentage of HRHPV14-positive LSIL diagnoses (presented as a decimal) relative to all LSIL diagnoses (lower curve), and the corresponding trend curves. The lowest cumulative percentage of LSIL diagnoses was 1.51% and the highest was 2.04%, and all values were between the 25th and 50th percentile reported for the SurePath LBC preparation. The cumulative percentage of HRHPV-positive LSIL for the entire study was 1.57% of all LBC diagnoses, and for LSIL diagnoses it was 77.17%.

**Figure 3:**
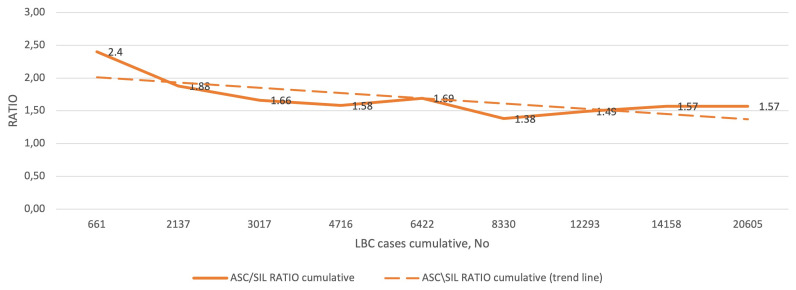
QC results for ASC/SIL in the study group (1–9 rounds). Abbreviations: QC, quality control; ASC, atypical squamous cells; SIL, squamous intraepithelial lesion; LBC, liquid-based cytology.

Figure 3 shows the cumulative ratio of ASC to SIL diagnoses, and the corresponding trend curve in the subsequent QA/QC rounds. The lowest ASC/SIL ratio was 1.38 and the highest was 2.4, and all values were between the 25th and 75th percentile (most below 1.8, i.e., between the 25th and 50th percentile) reported for the SurePath LBC preparation. The cumulative ASC/SIL ratio for the entire study was 1.57.

A summary of the complete results of the last QC round of the study is presented in Table 11. The PPV for positive (abnormal) LBC results in our study achieved 69.46%.

## 4. Discussion

In this aged-stratified study, data from private-based, opportunistic cervical cancer screening, including LBC, HRHPV14, and p16/Ki67 dual staining tests results, and their reporting rates with histologic correlations, were analyzed. Our investigation covered a total of 30.066 LBS screening tests assessed in a 5-year period in a Polish urban area population, and were highly consistent with other data, including ASCCP-sponsored US studies, data from non-US laboratories accredited by CAP, and with available data from Western European countries. Our experience with combinations of screening tests results provides the baseline data in cervical cancer screening in Poland, and when considering p16/Ki67 test results analysis, it also complements international data. Indirectly, this study demonstrates the applicability of the 2019 ASCCP risk-based guidelines for abnormal cervical cancer screening test results and cancer precursors outside the US. In the conditions of an ineffective public funds-based organized cervical cancer prevention program, private-based screening can effectively carry out screening, significantly supporting the national public system.

The HRHPV prevalence in women with normal cytology ranges from 6.6% to 22.9%, depending on geographic location. The estimated worldwide HPV DNA average rate is 10%. In our HPV study, the prevalence of HRHPV14 in women with NILM cytology results was 8.4% and is the closest to the levels reported by Northern European countries [29,30]. In the analysis of our results, we included American, Asian, and European population data as the comparators. The similarities to the HRHPV14-positivity of our results in all categories of abnormal cytological diagnoses were noted. Our HRHPV14-positivity rate of 48.9% for ASC-US cytology cases was within the levels reported for the US conditions, including CAP (47.73%) [31] and the Kaiser Permanente Northern California Foundation (48.39%) [17]. A meta-analysis of Arbyn et al. showed an average HRHPV-positivity rate for ASC-US cytology of 43.0%, but it should be remembered that slightly lower levels compared to ours in this case might be associated with the use of a different HRHPV test, which detects less high-risk types [32] than in our study. Meanwhile, the data from large-scale research carried out by CAP-accredited Chinese laboratories reports 48.7–54.7% for ASC-US HRHPV-positive cases [33,34]. Our results also demonstrated similar positive HRHPV rates in women with LSIL (77.2%) as reported by American (74.4–85.3%) [35,36], other CAP-accredited laboratories outside the US (75.2–79.2%) [34,37], and Western European countries (76.0%) [32]. Similarities in HRHPV-positivity rates for major cervical screening abnormalities encompassing ASC-H, HSIL, and AGC (84.6%; 90.7% and 26.7%, respectively) also were noted-for American reports and other CAP-accredited laboratories outside the US (78.07%; 88.1–94.78% and 16.47–27.78%, respectively) [36,38]. An exception is the HRHPV-positive LSIL reporting rate, with higher levels (85.3%) compared to our results (77.2%) [35]. There are several potential reasons for the differences, such as the wider age range in our study, including low HPV prevalent age groups, the heterogeneity of patients, and/or the different HPV test used.

The reliable comparison of cytology and HRHPV-positivity reporting rates, along with the histology detection rates with some large randomized Western European studies, was impossible due to the use of conventional cytological preparation, different cytologic classifications, combining cytological diagnoses into groups, or due to narrower age ranges analyzed when compared to our study. The reliable comparison between our study, in terms of HRHPV-positivity and cytology results, with European data from the study of the New Technologies for Cervical Cancer screening (NTCC) Working Group (published in Lancet Oncology [39]), where HRHPV-positivity in 9.56% of NILM was found, 45.95% of ASC-US, 72.05% of LSIL, and 92.11% of HSIL cytology, shows results all reporting rates similar to our own results.

The results of our study were highly consistent with large scale, ASCCP-sponsored investigations aimed at giving preliminary risk estimates for future guidelines. This high compliance rate has a particular relevance for the Polish interim guidelines for cervical cancer screening published in March 2021, as one of recommended screening approach is management according to the 2019 ASCCP-risk based guidelines. It might be possible that the introduction of the 2019 ASCCP guidelines in other Central-Eastern European countries with economic and cultural environments similar to that of Poland with conditions for proper QA/QC for gynecologic cytopathology.

The second goal of our investigation was to compare the obtained cytologic reporting rates with the data reported by CAP for the evaluation of gynecological cytopathology in percentile distributions. The analysis showed that our study results are within the ranges reported by the CAP-accredited laboratories, which confirms the possibility of effective application of the CAP cytopathology benchmarks in other geographic locations, and might substantiate the applicability of the 2019 ASCCP risk-based guidelines in Polish conditions. For the comparative analysis of cervical cytology reporting rates, we used the available benchmarking data reported by laboratories participating in the CAP accreditation program, after also implementing of the Bethesda 2014 system [21]. The distribution reporting rates for our LBC results were between the 25th and 50th percentiles for the vast majority cytologic categories. The percentages for our cytological diagnoses of ASC-US, LSIL, ASC-H, HSIL, and AGC were 3.35%, 2.04%, 0.22%, 0.24%, and 0.11%, respectively. The reporting rate in the percentile distribution for unsatisfactory cytology results (mostly too low cellularity) was 0.17%, and also was between the 25th and 50th percentile, as well as the ASC-SIL ratio, which was 1.56. The analysis of our cytopathological evaluation trends clearly suggests that the increase in the number of abnormal cytological diagnoses of ASC-US and LSIL is associated with the increasing percentage of cotesting results in the study group, and thus with the growing share of informed cytology evaluation in all HRHPV-positive cases, which is consistent with the important conclusions of NTCC group from their randomized controlled trial [40]. The PPV of the abnormal LBC results (ASC-US+) in our study was 69.46%, slightly below the range achieved by American laboratories (71–94%) [22]. However, it should be remembered that these ranges come from the work published in 1996, when surveillance management was recommended for patients with ASC-US and indications for a colposcopy were different without the HRHPV status analysis. In addition, colposcopy protocols at that time allowed for biopsy much less frequently, hence the availability of histology reports was lower, and thus potentially the number of false positives was also lower. Independently, the heterogeneity of the studied groups may be a factor determining the PPV level. Comparative data for the Polish population in that matter are lacking.

The histopathologic examination results demonstrated that a substantial number of our patients (28.3%) with NILM cytology HRHPV14-positive findings had HSIL (CIN2+), including 20.0% that were HSIL (CIN2). The histopathological results presented in other studies in the group of NILM HRHPV-positive patients differ significantly from 5.4% to 25.24%, with our histopathologic reporting rates similar to the ASCCP studies. Among the NILM HPV-negative cases, we found no HSIL (CIN2+) cases. The findings of our study support the HPV-based strategy (primary HPV or cotesting) in the Polish screening model and/or the need of a further triage option. In HRHPV14-positive cases with abnormal LBC diagnoses, we also did not find differences in the histopathological results compared to CAP certified laboratories outside the US, with immediate referrals for a colposcopy with a biopsy conducted.

Incorporating p16/Ki67 dual-stained cytology in our study provides Polish data on the proposed new tool for HSIL-risk detection, registered by the FDA as an alternative to cytology-based triage in HPV-based models [15,41]. The p16/Ki67 triage (i.e., in HRHPV-positive women with cytological NILM who had cotesting) increases the effectiveness of HSIL detection, which was also confirmed by our study–10.3% women in this group were p16/Ki67-positive, which allowed the detection of an additional 17 cases of HSIL (CIN2+), 19.54% of all detected HSIL (CIN2+). A total of 1130 p16/Ki67 tests were performed, of which 30% were positive, regardless of the age group and other screening tests results. Among the abnormal cytological diagnoses, the following percentages of p16/Ki67-positive cases was found: 30.0% for ASC-US; 35.2% for LSIL; 87.1% for ASC-H; 91.2% for HSIL; and 62.5% for AGC, which correlated with previous publications [42,43,44]. Similarities with other reports [13,43] were also observed in virologic-immunocytochemical-histologic correlations. Among the HRHPV-positive, p16/Ki67-positive with negative biopsy results, 39.6% of p16/Ki67-positive cases were found; with histologic LSIL (CIN1), 54.3%; and with histologic HSIL (CIN2 and CIN3), 90.1%. It should be pointed, that evaluation of p16/Ki67 dual-staining diagnostic value is the subject of another study of our group.

This analysis was also performed to create a baseline for the formation of an appropriate management method in cervical cancer screening for the Polish population, and to achieve the fullest data possible on the Polish private-based secondary cervical cancer prevention, which is characterized by a high market share of private medical services. Most Polish women declared participation in private opportunistic cervical cancer screening [45], whilst population coverage by the population-based national screening system is very low [6,7]. This situation is far from the screening effectiveness requirements, which clearly indicates the need for urgent changes. The European guidelines indicate an advantage of the population-based organized screening over the opportunistic one. However, a well-designed population-based program may not achieve the effectiveness parameters if it does not include local conditions [4,7]–one size does not fit all. Women’s preferences should be taken into account in the building of an effective model of secondary cervical cancer prevention in Poland and other countries with a similar history, screening tradition, and share of the private sector. Currently, as part of publicly funded care, the cytology-based opportunistic model only applies for women aged 25–59 in 3-year intervals [5]. The response to the limitations of Polish screening, also resulting from pandemic screening tests reduction, are the interim guidelines for cervical cancer screening, which approved the 2019 ASCCP Risk-Based Management as one of the possible screening options [18]. However, the application of the 2019 ASCCP guidelines to Polish conditions requires the fulfillment of basic conditions, including, in particular, the availability of validated diagnostic media, cytologic liquid-based preparation, HRHPV tests, standardized colposcopy, pathology infrastructure, and follow-up capabilities.

The strengths of the study include: (1) this is the first study analyzing such a large number of LBS results in Poland and Central Eastern Europe countries; (2) the large age range of the analyzed population; (3) our study provides insight into the screening tests results performed in the private-based opportunistic cervical cancer screening; (4) this is one of the largest studies presenting cytologic-virologic-immunocytochemical-histologic correlations in cervical cancer screening; (5) the time elapsed from the abnormal initial screening result and a colposcopy with a biopsy not exceeding 3 months, for an immediate histologic correlation; (6) the study shows the efficacy of QA/QC protocols for gynecologic cyto- and histopathology introduced outside of the laboratory. The limitations of the study include: (1) it is a post-hoc analysis; (2) not all patients with abnormal cytology obtained in cotesting, or primary cytology with reflex HRHPV14 testing, who had indications, decided to undergo a colposcopy with a biopsy at the Center; (3) the gynecological pathologist employed by the Center was controlled with internal QA/QC tools used outside of the laboratory performing the cytological and histopathological preparation; (4) the study showed a slightly lower positive predictive value in cytology for histologic HSIL detection (69.46%) than that reported by CAP. Due to different colposcopic protocols and/or different histologic terminology and/or the lack of p16 stain used in cervical histologic specimens, the results of colposcopic biopsies performed outside the Center were not included in the study.

## 5. Conclusions

In conclusion, the analysis of LBC, HRHPV14, and p16/Ki67 dual-staining reporting rates with histologic correlations presented in this age-stratified study on private opportunistic cervical cancer screening, the first on such a large scale in Poland and other countries of the region, demonstrates the possibility of the effective application of a private-based model in cervical cancer prevention. This is particularly relevant in light of the ineffectiveness of the organized population-based program due to low screening coverage. The data provided in our study can give a valuable benchmark, and might significantly fill the gap in Polish cervical cancer screening, not only in LBC and HRHPV14 testing, but also in p16/Ki67 dual staining. This study also showed that the new 2019 ASCCP risk-based guidelines can be reliably introduced outside the US, including in Polish private-based opportunistic secondary cervical cancer prevention, on the condition of adequate QA/QC gynecological cytopathology and colposcopy protocols being provided.

## Figures and Tables

**Table 1:** Selected QA/QC tools used in CQI protocol-groups of positive cytologic-immunocytochemical-histologic correlations with modification for the study *.

LBC, P16/Ki67 Diagnosis	Postcolposcopy Biopsy Result
LSIL ASC-US HRHPV14+ve HSIL SIL AIS CA p16/Ki67-positive	LSIL (CIN1) HSIL (CIN2) HSIL (CIN3) HSIL unspecified AIS CA

* Each combination of cytology results in the left column with histology results in the right column is a positive correlation. Abbreviations: QA/QC, quality assessment and quality control; CQI, continuous quality improvement; LBC, liquid-based cytology; LSIL, p16/Ki67, p16/Ki67 dual staining test; low-grade squamous intraepithelial lesion; HSIL, high-grade squamous intraepithelial lesion; SIL, squamous intraepithelial lesion; AIS, adenocarcinoma in situ; CA, cancer of the cervix; LSIL (CIN1), histologic low-grade squamous intraepithelial lesion; HSIL (CIN2), histologic high-grade squamous intraepithelial lesion with a quantification of cervical intraepithelial neoplasia in grade 2; HSIL (CIN3), histologic high-grade squamous intraepithelial lesion with a quantification of cervical intraepithelial neoplasia in grade 3; +ve, -positive.

**Table 2:** Selected QA/QC tools used in CQI protocol-groups of negative cytologic-immunocytochemical-histologic correlations with modification for the study *.

LBC, P16/Ki67 Diagnosis	Postcolposcopy Biopsy Result
LSIL ASC-US HRHPV14+ve HSIL SIL AIS CA p16/Ki67-positive	negative
NILM p16/Ki67-negative	LSIL (CIN1) HSIL (CIN2) HSIL (CIN3) HSIL unspecified AIS CA

* Each combination of cytology results in the left column with histology results in the right column is a negative correlation. Abbreviations: QA/QC, quality assessment and quality control; CQI, continuous quality improvement; LBC, liquid-based cytology; LSIL, p16/Ki67, p16/Ki67 dual staining test; low-grade squamous intraepithelial lesion; HSIL, high-grade squamous intraepithelial lesion; SIL, squamous intraepithelial lesion; AIS, adenocarcinoma in situ; CA, cancer of the cervix; LSIL (CIN1), histologic low-grade squamous intraepithelial lesion; HSIL (CIN2), histologic high-grade squamous intraepithelial lesion with a quantification of cervical intraepithelial neoplasia in grade 2; HSIL (CIN3), histologic high-grade squamous intraepithelial lesion with a quantification of cervical intraepithelial neoplasia in grade 3; +ve, -positive.

**Table 3:** Numbers of LBC, HRHPV14, and p16/Ki67 tests by year between August 2015 and July 2020.

Year	LBC, No.	HRHPV14, No. (%) ^1^	p16/Ki67, No. (%) ^2^	Total, No.
2015 (August–December)	2299	453 (19.7)	141 (6.1)	2893
2016	5131	1206 (23.5)	192 (3.7)	6529
2017	4475	1305 (29.2)	239 (5.3)	6019
2018	3833	2056 (53.6)	241 (6.3)	6130
2019	3411	2412 (70.7)	227 (6.7)	6050
2020 (January–July)	1456	899 (61.7)	90 (6.2)	2445
Total	20,605	8331 (40.4)	1130 (5.5)	30,066

Abbreviations: LBC, liquid-based cytology; HRHPV14, 14 high-risk types human papillomavirus test; p16/Ki67, p16/Ki67 dual staining test; ^1^ % LBC with HRHPV14 results; ^2^ % LBC results with p16/Ki67 results.

**Table 4:** Age-stratified LBS tests reporting rates.

Age, Years	LBC, No.	HRHPV14, No.	p16/Ki67, No.	Total, No.
<21	226	40	16	282
21–24	1224	373	98	1695
25–29	2410	908	212	3530
30–39	7285	3166	466	10,917
40–49	4613	2248	219	7080
50–59	2481	939	66	3486
60–65	1384	424	36	1844
≥66	982	233	17	1232
Total	20,605	8331	1130	30,066

Abbreviations: LBS, liquid-based screening; LBC, liquid-based cytology; HRHPV14, 14 high-risk types human papillomavirus test; p16/Ki67, p16/Ki67 dual staining test.

**Table 5:** Overall NILM, ASC-US, LSIL, ASC-H, HSIL, and AGC reporting rates in the study group with relevant HRHPV14 and p16/Ki67 results.

LBC Test Result	Cases, No (%) ^1^	HRHPV14 Results, No (%) ^2^	HRHPV14+ve Results, No (%) ^3^	P16/Ki67 Results, No (%) ^2^	P16/Ki67+ve Results, No (%) ^4^
NILM	19,338 (93.9)	7283 (37.7)	612 (8.4)	458 (2.4)	80 (17.4)
ASC-US	690 (3.4)	571 (82.8)	279 (48.9)	323 (46.8)	97 (30.0)
LSIL	420 (2.0)	368 (87.6)	284 (77.2)	273 (65.0)	96 (35.2)
ASC-H	46 (0.22)	39 (84.8)	33 (84.6)	31 (67.4)	27 (87.1)
HSIL	49 (0.24)	43 (87.8)	39 (90.7)	34 (69.4)	31 (91.2)
AGC	23 (0.11)	15 (65.2)	4 (26.7)	8 (34.8)	5 (62.5)
ADC	2 (0.01)	1 (50.0)	0 (0.0)	1 (50.0)	1 (100.0)
SCC	2 (0.01)	0 (0.0)	0 (0.0)	1 (50.0)	1 (100.0)
unsatisfactory	35 (0.17)	11 (31.4)	5 (45.5)	1 (2.9)	1 (100.0)
Total	20,605	8331 (40.4)	1256 (15.1)	1130 (5.5)	339 (30.0)

Abbreviations: NILM, negative for intraepithelial lesion or malignancy; ASC-US, atypical squamous cells of undetermined significance; LSIL, low-grade squamous intraepithelial lesion; ASC-H, atypical squamous cells-cannot excluded HSIL; HSIL, high-grade squamous intraepithelial lesion; AGC, atypical glandular cells; ADC, adenocarcinoma; SCC, squamous cell carcinoma; LBC, liquid-based cytology; HRHPV14, 14 high-risk types human papillomavirus test; +ve, positive; p16/Ki67, p16/Ki67 dual staining test; ^1^ % of all LBC cases; ^2^ % of results defined in the first column; ^3^ % results defined in the first column with HRHPV14 results; ^4^ % results defined in the first column with p16/Ki67 results.

**Table 6:** Age-stratified NILM, ASC-US, LSIL, ASC-H, HSIL, and AGC reporting rates in the study group.

Group No	Age, Years	NILM, No (%)	ASC-US No (%)	LSIL No (%)	ASC-H No (%)	HSIL No (%)	AGC No (%)
1.	<21	204 (90.3)	6 (2.7)	15 (6.6)	0 (0.0)	0 (0.0)	0 (0.0)
2.	21–24	1116 (91.2)	45 (3.7)	56 (4.6)	3 (0.25)	2 (0.16)	0 (0.0)
3.	25–29	2194 (91.0)	99 (4.1)	91 (3.8)	7 (0.29)	8 (0.33)	3 (0.13)
4.	30–39	6835 (93.8)	241 (3.3)	153 (2.1)	18 (0.25)	23 (0.32)	2 (0.03)
5.	40–49	4333 (94.0)	168 (3.6)	71 (1.5)	16 (0.35)	9 (0.20)	12 (0.26)
6.	50–59	2386 (96.2)	66 (2.7)	18 (0.73)	1 (0.04)	1 (0.04)	4 (0.16)
7.	60–65	1337 (96.6)	30 (2.2)	8 (0.58)	1 (0.07)	5 (0.36)	1 (0.07)
8.	≥66	933 (95.0)	35 (3.6)	8 (0.82)	0 (0.0)	1 (0.10)	1 (0.10)
	Total	19,338 (93.9)	690 (3.4)	420 (2.0)	46 (0.22)	49 (0.24)	23 (0.11)

Abbreviations: NILM, negative for intraepithelial lesion or malignancy; ASC-US, atypical squamous cells of undetermined significance; LSIL, low-grade squamous intraepithelial lesion; ASC-H, atypical squamous cells-cannot excluded HSIL; HSIL, high-grade squamous intraepithelial lesion; AGC, atypical glandular cells.

**Table 7:** The histologic correlation for LBC (CHC).

	No (% ^1^/% ^2^)	Histology Result, No (%) ^3^			
LBC Result	Total Biopsy Results	Negative	LSIL (CIN1)	HSIL (CIN2)	HSIL (CIN3+)
NILM	62 (0.32/14.9)	37 (59.7)	8 (12.9)	12 (19.4)	5 (8.1)
ASC-US	153 (22.2/36.9)	83 (54.3)	47 (30.7)	14 (9.2)	9 (5.9)
LSIL	139 (33.1/33.5)	47 (33.8)	71 (51.1)	16 (11.5)	5 (3.6)
ASC-H	25 (54.4/6.0)	8 (32.0)	6 (24.0)	5 (20.0)	6 (24.0)
HSIL	26 (53.1/6.3)	4 (15.4)	2 (7.7)	6 (23.1)	14 (53.9)
AGC	10 (43.5/2.4)	9 (90.0)	0 (0.0)	0 (0.0)	1 (10.0)
SCC/AIS	0 (0.0/0.0)	0 (0.0)	0 (0.0)	0 (0.0)	0 (0.0)
Total	415 (2.0/100.0)	188 (45.3)	134 (32.3)	53 (12.8)	40 (9.6)

Abbreviations: LBC, liquid-based cytology; CHC, cyto-histologic correlations; NILM, negative for intraepithelial lesion or malignancy; ASC-US, atypical squamous cells of undetermined significance; LSIL, low-grade squamous intraepithelial lesion; ASC-H, atypical squamous cells-cannot excluded HSIL; HSIL, high-grade squamous intraepithelial lesion; AGC, atypical glandular cells; SCC, squamous cell carcinoma; AIS, adenocarcinoma in situ; LSIL (CIN1), histologic low-grade squamous intraepithelial lesion; HSIL (CIN2), histologic high-grade squamous intraepithelial lesion with a quantification of cervical intraepithelial neoplasia in grade 2; HSIL (CIN3+), histologic high-grade squamous intraepithelial lesion with a quantification of cervical intraepithelial neoplasia in grade 3 or worse; ^1^ % of results defined in the first column; ^2^ % of all biopsy results; ^3^ % of total biopsy results for the LBC result defined in the first column.

**Table 8:** The histologic correlation for LBC and HRHPV14 results (CVHC).

	No (%) ^1^	Histology Result, No (%) ^2^			
LBC Result	TBR with HPV Results	Negative	LSIL (CIN1)	HSIL (CIN2)	HSIL (CIN3+)
NILM	61 (8.4)	36 (59.0)	8 (13.1)	12 (19.7)	5 (8.2)
HRHPV14+ve	60 (8.2)	35 (58.3)	8 (13.3)	12 (20.0)	5 (8.3)
HRHPV14−ve	1 (0.01)	1 (100.0)	0 (0.0)	0 (0.0)	0 (0.0)
ASC-US	147 (25.7)	82 (55.8)	42 (28.6)	14 (9.5)	9 (6.1)
HRHPV14+ve	139 (24.3)	75 (54.0)	41 (29.5)	14 (10.1)	9 (6.5)
HRHPV14−ve	8 (1.4)	7 (87.5)	1 (12.5)	0 (0.0)	0 (0.0)
LSIL	136 (37.0)	45 (33.1)	71 (52.2)	15 (11.0)	5 (3.7)
HRHPV14+ve	131 (35.6)	42 (32.1)	69 (52.7)	15 (11.5)	5 (3.8)
HRHPV14−ve	5 (1.4)	3 (60.0)	2 (40.0)	0 (0.0)	0 (0.0)
ASC-H	25 (64.1)	8 (32.0)	6 (24.0)	5 (20.0)	6 (24.0)
HRHPV14+ve	22 (56.4)	6 (27.3)	5 (22.7)	5 (22.7)	6 (27.3)
HRHPV14−ve	3 (7.7)	2 (66.7)	1 (33.3)	0 (0.0)	0 (0.0)
HSIL	25 (58.1)	4 (16.0)	2 (8.0)	6 (24.0)	13 (52.0)
HRHPV14+ve	23 (53.5)	4 (17.4)	2 (8.7)	5 (21.7)	12 (52.2)
HRHPV14−ve	2 (4.7)	0 (0.0)	0 (0.0)	1 (50.0)	1 (50.0)
AGC	8 (53.3)	7 (87.5)	0 (0.0)	0 (0.0)	1 (12.5)
HRHPV14+ve	2 (13.3)	1 (50.0)	0 (0.0)	0 (0.0)	1 (50.0)
HRHPV14−ve	6 (40.0)	6 (100.0)	0 (0.0)	0 (0.0)	0 (0.0)
Total	402 (4.8)	182 (45.3)	129 (32.1)	52 (12.9)	39 (9.7)
HRHPV14+ve	377 (4.5)	163 (43.2)	125 (33.2)	51 (13.5)	38 (10.1)
HRHPV14−ve	25 (0.30)	19 (76.0)	4 (16.0)	1 (4.0)	1 (40)

Abbreviations: LBC, liquid-based cytology; HRHPV14, 14 high-risk types human papillomavirus test; +ve, positive; −ve, negative; CVHC, cyto-viro-histologic correlations; TBR, total biopsy results; NILM, negative for intraepithelial lesion or malignancy; ASC-US, atypical squamous cells of undetermined significance; LSIL, low-grade squamous intraepithelial lesion; ASC-H, atypical squamous cells-cannot excluded HSIL; HSIL, high-grade squamous intraepithelial lesion; AGC, atypical glandular cells; LSIL (CIN1), histologic low-grade squamous intraepithelial lesion; HSIL (CIN2), histologic high-grade squamous intraepithelial lesion with a quantification of cervical intraepithelial neoplasia in grade 2; HSIL (CIN3+), histologic high-grade squamous intraepithelial lesion with a quantification of cervical intraepithelial neoplasia in grade 3 or worse; ^1^ % of results defined in the first column; ^2^ % of total biopsy results for the LBC result and HRHPV14 status defined in the first column.

**Table 9:** The histologic correlation for LBC, HRHPV14, and p16/Ki67 results (CVICHC).

	No (%) ^1^	Histology Result, No (%) ^2^			
LBC Result	TBR with HRHPV14 + P16/Ki67 Results	Negative	LSIL (CIN1)	HSIL (CIN2)	HSIL (CIN3+)
NILM	59 (12.9)	34 (57.6)	8 (13.6)	12 (20.3)	5 (8.5)
HPV+ve	58 (12.7)	33 (56.9)	8 (13.8)	12 (20.7)	5 (8.6)
p16/Ki67 (+)	47 (10.3)	23 (48.9)	7 (14.9)	12 (25.5)	5 (10.6)
p16/Ki67 (−)	11 (2.4)	10 (90.9)	1 (9.1)	0 (0.0)	0 (0.0)
HPV−ve	1 (0.22)	1 (100.0)	0 (0.0)	0 (0.0)	0 (0.0)
p16/Ki67 (+)	1 (0.22)	1 (100.0)	0 (0.0)	0 (0.0)	0 (0.0)
p16/Ki67 (−)	0 (0.0)	0 (0.0)	0 (0.0)	0 (0.0)	0 (0.0)
ASC-US/LSIL	268 (45.0)	118 (44.0)	107 (39.9)	29 (10.8)	14 (5.2)
HPV+ve	252 (42.3)	107 (42.5)	102 (40.5)	29 (11.5)	14 (5.6)
p16/Ki67 (+)	126 (21.1)	30 (23.8)	55 (43.7)	27 (21.4)	14 (11.1)
p16/Ki67 (−)	126 (21.1)	77 (61.1)	47 (37.3)	2 (1.6)	0 (0.0)
HPV−ve	16 (2.7)	11 (68.8)	5 (31.3)	0 (0.0)	0 (0.0)
p16/Ki67 (+)	12 (2.0)	8 (66.7)	4 (33.3)	0 (0.0)	0 (0.0)
p16/Ki67 (−)	4 (0.67)	3 (75.0)	1 (25.0)	0 (0.0)	0 (0.0)
ASC-H+	48 (65.8)	14 (29.2)	7 (14.6)	10 (20.8)	17 (35.4)
HPV+ve	42 (57.5)	11 (26.2)	6 (14.3)	9 (21.4)	16 (38.1)
p16/Ki67 (+)	35 (48.0)	9 (25.7)	4 (11.4)	7 (20.0)	15 (42.9)
p16/Ki67 (−)	7 (9.6)	2 (28.6)	2 (28.6)	2 (28.6)	1 (14.3)
HPV−ve	6 (8.2)	3 (50.0)	1 (16.7)	1 (16.7)	1 (16.7)
p16/Ki67 (+)	4 (5.5)	1 (25.0)	1 (25.0)	1 (25.0)	1 (25.0)
p16/Ki67 (−)	2 (2.7)	2 (100.0)	0 (0.0)	0 (0.0)	0 (0.0)
Total	375 (33.3)	166 (44.3)	122 (32.5)	51 (13.6)	36 (9.6)
HPV+ve	352 (31,.2)	151 (42.9)	116 (33.0)	50 (14.2)	35 (9.9)
p16/Ki67 (+)	208 (18.5)	62 (29.8)	66 (31.7)	46 (22.1)	34 (16.3)
p16/Ki67 (−)	144 (12.8)	89 (61.8)	50 (34.7)	4 (2.8)	1 (0.69)
HPV−ve	23 (2.0)	15 (65.2)	6 (26.1)	1 (4.4)	1 (4.4)
p16/Ki67 (+)	17 (1.5)	10 (58.8)	5 (29.4)	1 (5.9)	1 (5.9)
p16/Ki67 (−)	6 (0.53)	5 (83.3)	1 (16.7)	0 (0.0)	0 (0.0)

Abbreviations: LBC, liquid-based cytology; HRHPV14, 14 high-risk types human papillomavirus test; p16/Ki67, p16/Ki67 dual staining test; +ve, positive; −ve, negative; CVICHC, cyto-virologic-immunocytochemical-histologic correlations; TBR, total biopsy results; NILM, negative for intraepithelial lesion or malignancy; ASC-US, atypical squamous cells of undetermined significance; LSIL, low-grade squamous intraepithelial lesion; ASC-H+, cytology results: ASC-H (atypical squamous cells-cannot excluded HSIL), HSIL (high-grade squamous intraepithelial lesion) or AGC (atypical glandular cells); LSIL (CIN1), histologic low-grade squamous intraepithelial lesion; HSIL (CIN2), histologic high-grade squamous intraepithelial lesion with a quantification of cervical intraepithelial neoplasia in grade 2; HSIL (CIN3+), histologic high-grade squamous intraepithelial lesion with a quantification of cervical intraepithelial neoplasia in grade 3 or worse; ^1^ % of results defined in the first column; ^2^ % of total biopsy results for the LBC result, HRHPV14 and p16/Ki67 status defined in the first column.

**Table 10:** Following QC rounds with cumulative number of LBC cases.

QC Round, No	QC Date	No of LBC Cases, Cumulative
1	September 2015	661
2	December 2015	2137
3	February 2016	3017
4	June 2016	4716
5	October 2016	6422
6	July 2017	8330
7	July 2018	12,293
8	July 2019	17,167
9	July 2020	20,605

Abbreviations: QC, quality control; LBC, liquid-based cytology.

**Table 11:** Cyto-virologic correlations (CVC) for the study population for the CQI protocol (round 9).

LBC Result	No (%) ^1^	HRHPV14 Results, No (%) ^2^	HRHPV14+ve, No (%) ^3^
NILM	19,338 (93.85)	7283 (37.66)	612 (8.40)
ASC-US	690 (3.35)	571 (82.75)	279 (48.86)
ASC-H	46 (0.22)	39 (84.78)	33 (84.62)
LSIL	420 (2.04)	368 (87.62)	284 (77.17)
HSIL	49 (0.24)	43 (87.76)	39 (90.70)
ASC/SIL	1.57	x	x
AGC	23 (0.11)	15 (65.22)	4 (26.67)
unsatisfactory	35 (0.17)	11 (31.43)	5 (45.45)
SCC	2 (0.01)	0 (0.00)	0 (0.00)
ADC	2 (0.01)	1 (50.00)	0 (0.00)
Total	20,605 (100.00)	8331 (40.43)	1256 (15.08)

Abbreviations: CVC, cyto-virologic correlations; CQI, continuous quality improvement; LBC, liquid-based cytology; HRHPV14, 14 high-risk types human papillomavirus test; +ve, positive; NILM, negative for intraepithelial lesion or malignancy; ASC-US, atypical squamous cells of undetermined significance; ASC-H, atypical squamous cells-cannot excluded HSIL; LSIL, low-grade squamous intraepithelial lesion; HSIL, high-grade squamous intraepithelial lesion; ASC/SIL, atypical squamous cells/squamous intraepithelial lesion ratio; AGC, atypical glandular cells; SCC, squamous cell carcinoma; ADC, adenocarcinoma; ^1^ % of total LBC results; ^2^ % of results defined in the first column; ^3^ % of results in the third column.

## Data Availability

The data presented in this study are available on request from the corresponding author.
